# Exploration of Prognostic Immune-Related Genes and lncRNAs Biomarkers in Kidney Renal Clear Cell Carcinoma and Its Crosstalk with Acute Kidney Injury

**DOI:** 10.1155/2022/6100187

**Published:** 2022-02-08

**Authors:** Chenxia Juan, Ye Zhu, Yan Zhou, Weiwei Zhu, Xufang Wang, Weiming He, Yan Chen

**Affiliations:** ^1^Department of Nephrology, Jiangsu Province Hospital of Chinese Medicine, Affiliated Hospital of Nanjing University of Chinese Medicine, Nanjing, Jiangsu, China; ^2^Department of Nephrology, Second Affiliated Hospital of Soochow University, Suzhou, Jiangsu, China; ^3^Department of Nephrology, Jiangsu Province Geriatric Hospital, Jiangsu Province Official Hospital, Nanjing, Jiangsu, China

## Abstract

Kidney renal clear cell carcinoma (KIRC) has a poor prognosis and a high death rate globally. Cancer prognosis is strongly linked to immune-related genes (IRGs), according to numerous research. We utilized KIRC RNA-seq data from the TCGA database to build a prognostic model incorporating seven immune-related (IR) lncRNAs, and we constructed the model using LASSO regression. Additionally, we calculated a risk score for each patient using a prognostic model that divided patients into high-risk and low-risk groups. The ESTIMATE and CIBERSORT methodologies were then used to analyze the differences in the tumor microenvironment of the two groups of patients. Finally, we predicted three small molecule drugs that may have potential therapeutic effects for high-risk patients. We combined the acute kidney injury dataset to obtain differential genes that may serve standard biological functions with two risk groups. Our study shows that the model we constructed for IR-lncRNAs has reliable predictive efficacy for patients with KIRC.

## 1. Introduction

Kidney renal clear cell carcinoma (KIRC) is a common tumor, accounting for 70–80% of all kidney tumors [1]. Every year, around 210,000 new cases of KIRC are diagnosed throughout the globe [2]. Despite significant progress in recent years in understanding the mechanisms and treatments of KIRC, the prognosis for KIRC patients remains poor [3]. For instance, the 5-year overall survival (OS) rate for stage I KIRC patients is around 80%–95%, whereas the 5-year OS rate for stage IV KIRC patients is less than 10% [4]. As a result, new biomarkers and therapy alternatives are urgently needed to assist doctors in identifying suitable treatment options and medicines and more correctly predicting the prognosis of KIRC patients.

Tumor immunity has attracted scientists' attention as a result of advances in the research of various cancers. Immune-related genes (IRGs) are thought to have a role in tumor growth and progression [5,6]. In various cancers, immunotherapy that targets PD-1 ligand (PD-L1)/programmed death 1 (PD-1) signalling, for example, has been demonstrated to increase antitumor immunity [7]. Furthermore, studies have shown that PD-L1 and PD-1 expressions in KIRC patients are higher and correlate with patient prognosis and that immune checkpoint inhibitor drugs provide better options for KIRC patients, such as improved OS and chemotherapy tolerance [8]. Tumor microenvironment (TME) research is also becoming more critical in the development of tumors [9]. The TME comprises extracellular matrix components, immune cells, stromal cells, and inflammatory mediators [10]. One of the primary cell types in the TME, immune infiltrating cells, is strongly associated with tumor therapeutic response [11]. Using immune-based prognostic markers in KIRC is a promising method, according to the results of these studies [12, 13]. Furthermore, the findings of this research might be utilized to investigate the underlying biological processes further and identify possible therapeutic medications that could help KIRC patients have a better prognosis.

Through transcription regulation, long noncoding RNAs (lncRNAs) have been shown to have a role in the creation and activation of a variety of immune cells, as well as tumor metastasis [14, 15]. Immune-related lncRNAs (IR-lncRNAs) can be used as markers to predict the prognosis of glioma patients [16]. It has been shown that creating IR-related models may predict the prognosis of patients with various kinds of cancers, including lung cancer, glioblastoma, and gastric cancer [17–19]. However, the prognostic significance of IRGs and IR-lncRNAs for KIRC is currently being researched. We analyzed the relevance of IRGs and IR-lncRNAs in predicting the prognosis of KIRC patients in this work. We used least absolute shrinkage and selection operator (LASSO) regression to develop a model incorporating seven IR-lncRNAs for predicting overall survival. Based on the model, risk scores were produced for each patient, and patients were split into low- and high-risk groups. The TME of the samples was then examined by utilizing ESTIMATE and CIBERSORT algorithms to calculate the quantity of stromal and immune cells in every sample. Finally, we identified three small-molecule medicines that might be used to treat individuals at high risk of KIRC. We also looked at the AKI dataset to see whether any genes had both interactions with KIRC. We found that the model we constructed consisting of IRGs and IR-lncRNA was able to predict the prognosis of KIRC patients.

## 2. Materials and Methods

### 2.1. Data Collection

We downloaded data from the TCGA (The Cancer Genome Atlas) database for 537 RNA-seq cases, containing clinical information of the cases. We downloaded the RNA-seq dataset containing 39 human kidney biopsy samples (AKI group) and 9 reference nephrectomy groups (REF) from GEO (Gene Expression Omnibus) (GSE139061). Clinical features of 537 samples are shown in Table 1. The latter study included patients with KIRC who had complete clinical data and RNA-seq data. ImmPort Shared Data provided us with a total of 2483 IRGs (Table S1).

### 2.2. WGCNA Network Construction

To discover modular genes associated with clinical features, we used the weighted gene coexpression network analysis (WGCNA) approach to construct a network [20]. The gene-gene association is determined for each pair in the first stage using gene coexpression similarity. The adjacency matrix and topological overlap matrix (TOM) are then constructed using “soft” criteria through the adjacency function. Using a hierarchical clustering of heterogeneity measure (1-TOM) method, each “gene module” is a set of genes with a high degree of topological overlap.

Pearson's technique was used to find correlations between modules and clinical variables to find clinically relevant modules. We then chose modules that had a strong association with prognostic characteristics.

### 2.3. Identification of Prognosis-Related IRG and IR-lncRNAs

All of the IRGs in the prognosis-related module were subjected to univariate Cox regression analysis, and only those with a *P* value < 0.05 were recognized as prognosis-related. We then used Pearson correlation analysis to determine the correlations between prognostic IRGs and all lncRNAs in KIRC patients; correlations more than 0.6 were designated as IR-lncRNAs. Finally, all IR-lncRNAs were submitted to univariate Cox regression, and lncRNAs with *P* < 0.001 were selected as prognostically relevant IR-lncRNAs.

### 2.4. Establishment of Prognostic Signature

We used LASSO Cox regression to analyze all prognosis-related IRG and IR-lncRNAs to construct a prognostic model. The following equation was used to obtain the model risk scores:

(1) $$\begin{array}{c} \text{Risk score}=\sum_{i=1}^{n}\beta i*xi, \end{array}$$

where *xi* is the gene expression value and *βi* reflects the coefficient produced from LASSO Cox regression analysis; based on the median values of the calculated risk scores, we divided the KIRC patients into two groups of high risk and low risk. To validate the model, we had three cohorts, the training set, the validation set (random 1 : 1 assignment), and the total cohort, which were analyzed using Kaplan-Meier's method. To validate the predictive efficacy of the model, we used time-dependent ROC curves, combined with clinical information for univariate and multifactorial analyses.

### 2.5. Immune Infiltration Analysis

To investigate the relationship between our predictive model and immune cell infiltration, we used the CIBERSORT method [21] to calculate the amount of 22 immune cells in each KIRC sample; we also used the Wilcoxon rank-sum test to compare the differences in immune cells between high- and low-risk groups and then used the K-M method to look at the relationship between immune cell infiltration and the OS of KIRC patients.

### 2.6. Pathway Enrichment Analysis and Identification of DEGs

We used the edgeR package to analyze the differentially expressed genes (DEGs) in the two groups. We used CMap, a database of small molecule drugs (https://portals.broadinstitute.org/cmap), set |log2FC| ≥ 1, and finally calculated an enrichment score. A positive score suggests that the drug may raise the risk of mortality in people with KIRC. A negative value implies that the drug may reduce the risk of mortality in individuals with KIRC. A drug with a negative score has the potential to be therapeutic. Using the PubChem database, we constructed two-dimensional structural maps of the prospective small molecule medicines (https://pubchem.ncbi.nlm.nih.gov/).

### 2.7. GSEA

We used gene set enrichment analysis to see whether there was a substantial difference in gene set enrichment between the high- and low-risk categories in the MSigDB collection (GSEA) [22]. For each study, 1,000 permutations of gene sets were done.

### 2.8. Statistical Analysis

We used *R* software (version 3.8.0) to perform statistical analysis. Risk scores and clinical features were examined using the *χ*^2^ test to see whether there was any correlation. The statistical significance of normally distributed variables was compared between the two groups using unpaired *t*-tests. All statistical tests were two-tailed, and statistical significance was defined as a value of *P* < 0.05.

## 3. Results

### 3.1. Construction of a Coexpression Network

**Figure 1:**
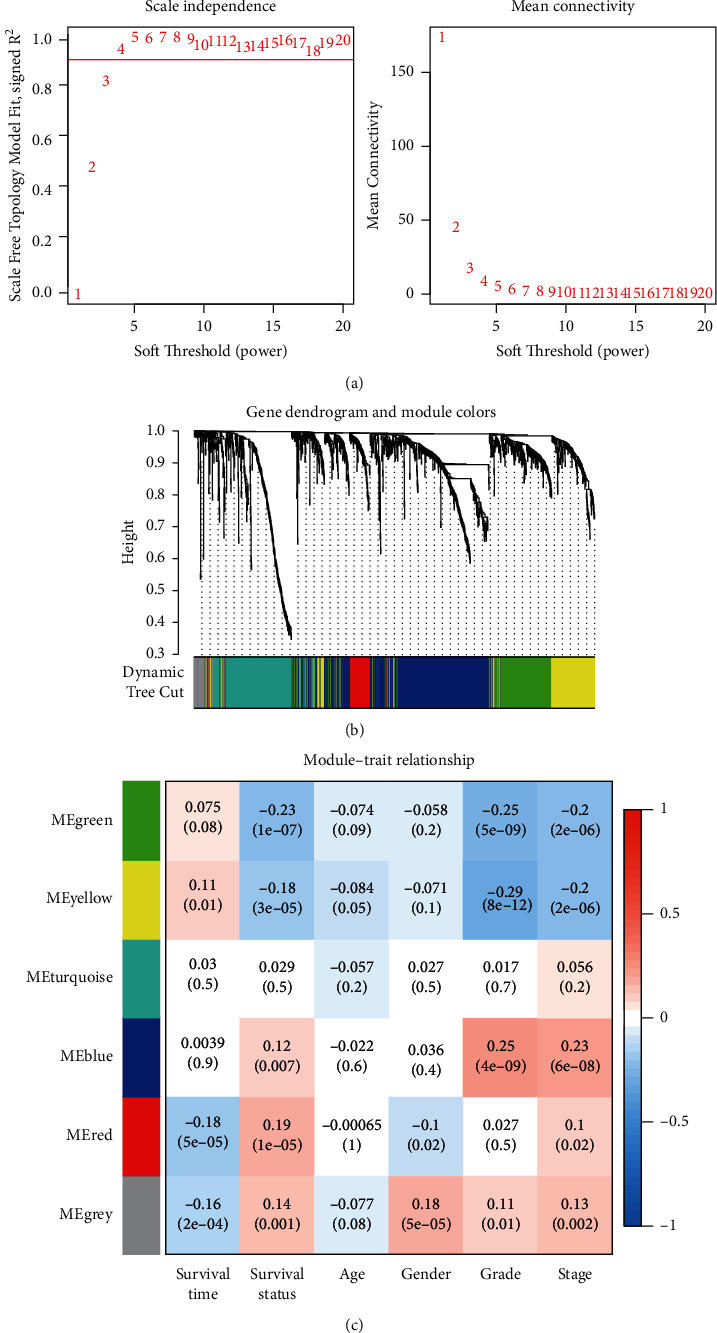
WGCNA network construction and module identification. (a) Soft thresholds determined. (b) WGCNA module identification and clustering dendrogram. (c) Matrix of eigengene values and clinical characteristics.

A total of 2483 IRGs from 539 KIRC samples were subjected to a WGCNA study. When the threshold value is set to 4, the gene network's connections form a scale-free network distribution (Figure 1(a)). Then we got six coexpressed modules in various colours (Figure 1(b)). Age, sex, grade, stage, overall survival, and survival status were used to determine the connection between modules and clinical features. The survival status of red and grey modules was discovered to be connected (Figure 1(c)). Consequently, the red and grey modules were selected as potential candidates for further investigation in the upcoming research.

### 3.2. Identification of Prognostic-Related IRGs and IR-lncRNAs

The follow-up survival study includes 526 samples with complete survival data. For all IRGs in the red (*n* = 74) and grey (*n* = 80) modules, a univariate Cox regression analysis was conducted (Tables S2 and S3), and 63 genes with *P* < 0.05 were identified as prognosis-associated IRGs (Table S4). Subsequently, correlations ≥0.6 were obtained for 313 IR-lncRNAs with prognostic IRGs. Also, 206 IR-lncRNAs had good predictive value with *P* < 0.001 in the one-way Cox regression analysis (Table S5).

### 3.3. Construction of a Predictive Model

**Figure 2:**
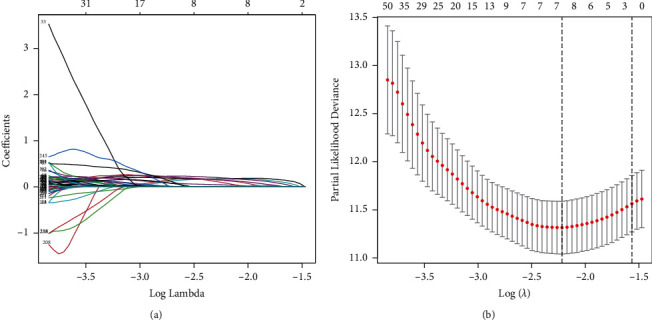
LASSO Cox regression identified 7 lncRNAs for model construction (a, b).

We used LASSO regression analysis to screen for efficient prognostic-related markers, and a prognostic model was constructed from the training set (Figure 2). The most helpful predictive biomarkers were discovered as DLGAP1-AS2, AC024060.2, SLBP-DT, DGUOK-AS1, MYG1-AS1, AC00578.1, and MELTF-AS1 (Table 2).

**Figure 3:**
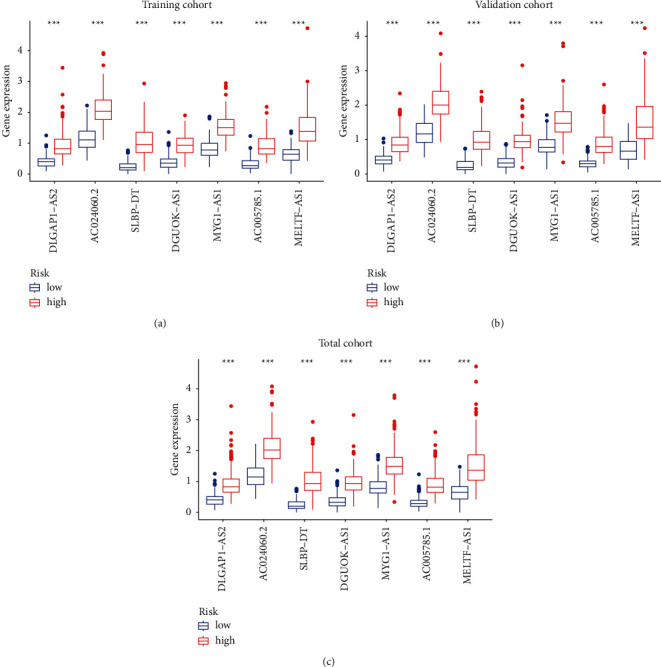
Expression levels of seven biomarkers in the training group (a), validation group (b), and total cohort (c) in the high-risk and low-risk group cohorts.

We calculated a risk score for each patient and then divided the patients into low-risk and high-risk groups by a median value of cut-off. The expression levels of biomarkers in each group were also analyzed (Figure 3).

### 3.4. Correlation between Signature and Clinicopathologic Characteristics

Age, gender, class, and N categorization did not vary substantially between the high-risk and low-risk groups in any of the cohorts, as shown in Table 3. The training group and the overall cohort, on the other hand, had substantially different stages, T classification, and M classification.

### 3.5. Prognostic Value of Signature for Assessing Overall Survival

**Figure 4:**
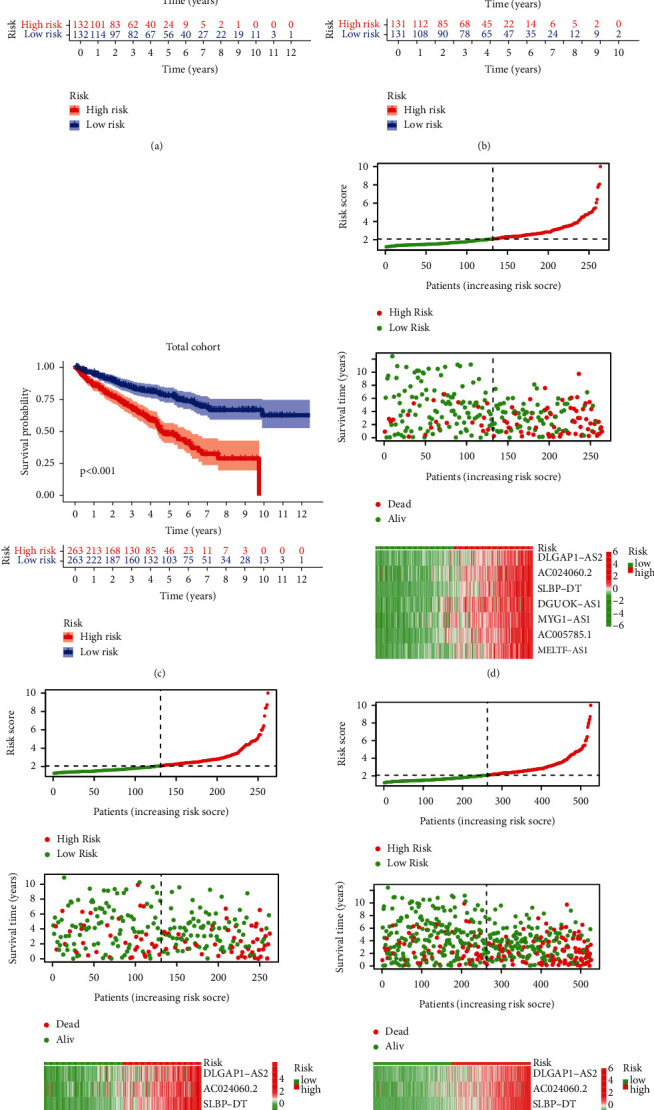
The predictive value of a model based on IR-lncRNAs. OS for high- and low-risk patients in the three cohorts (a–c) analyzed using the K-M method. Risk scores, survival status, and expression distribution of all seven lncRNAs in the prognostic model for patients in high- and low-risk groups were calculated in the three cohorts (d–f).

**Figure 5:**
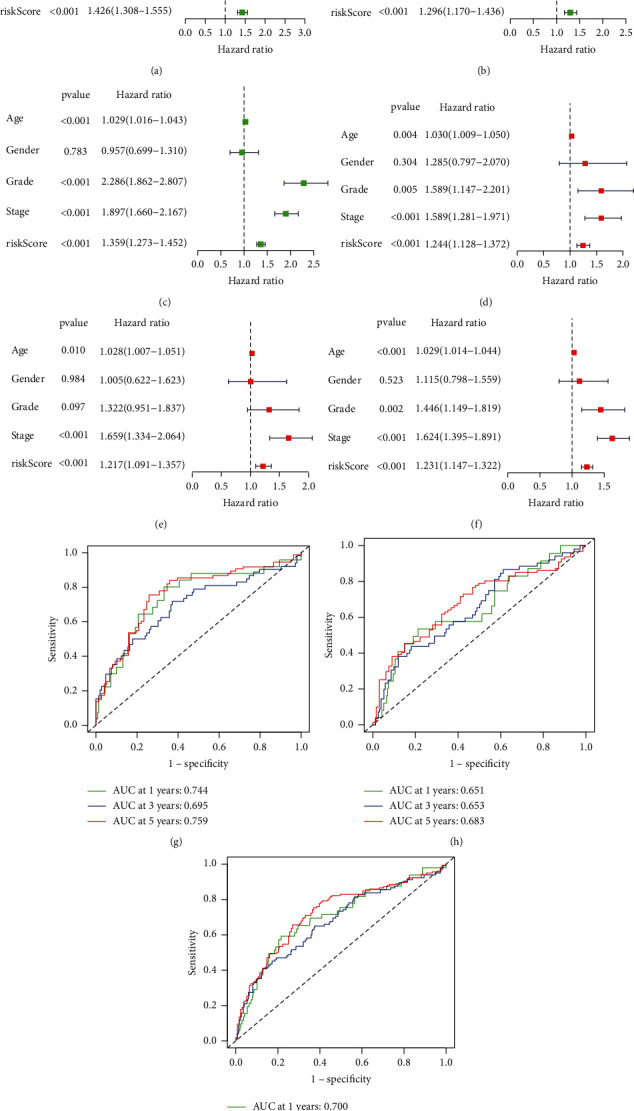
Prognostic value of the signature. Univariate and multivariate Cox analyses of predictive model in the training (a, d), validation (b, e), and total cohorts (c, f). AUC for 1-, 3-, and 5-year OS prediction for the prognostic models in three cohorts (g–i).

Patients in the high-risk group have a poorer prognosis, as indicated in Figures 4(a)–4(c). Patients' survival times fell as their risk score rose, as seen in Figures 4(d)–4(f), and more patients died in the high-risk group. The Kaplan-Meier test was used to confirm the model's prognostic usefulness further. Univariate Cox regression analysis supported the model's prognostic usefulness (Figures 5(a)–5(c)). In a multivariate analysis of the whole cohort, the risk score was an independent risk factor for KIRC patient OS (Figures 5(d)–5(f)). The AUC for OS at 1, 3, and 5 years was 0.744, 0.695, and 0.759 in training cohort; 0.651, 0.653, and 0.683 in validation cohort (Figure 5(h)); and 0.700, 0.675, and 0.723 in the total cohort (Figure 5(i)).

### 3.6. High-Risk Scores Were Related to Higher Immune Scores in KIRC Patients

**Figure 6:**
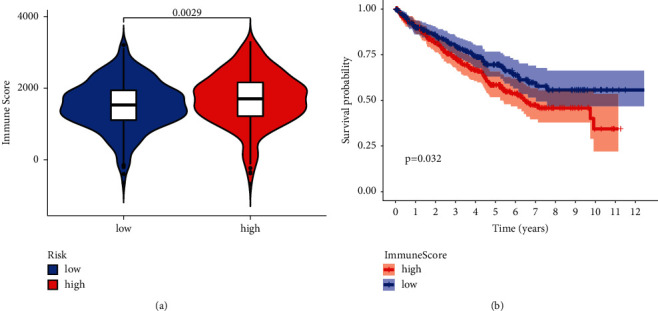
(a) High immune score in patients with high-risk score. (b) K-M analysis of OS between high and low immune scores.

As seen in Figure 6(a), individuals classified as high risk had significantly higher immunological ratings. Furthermore, patients with high immunological ratings were linked to a worse prognosis (Figure 6(b)).

### 3.7. The Landscape of Immune Infiltration in KIRC

**Figure 7:**
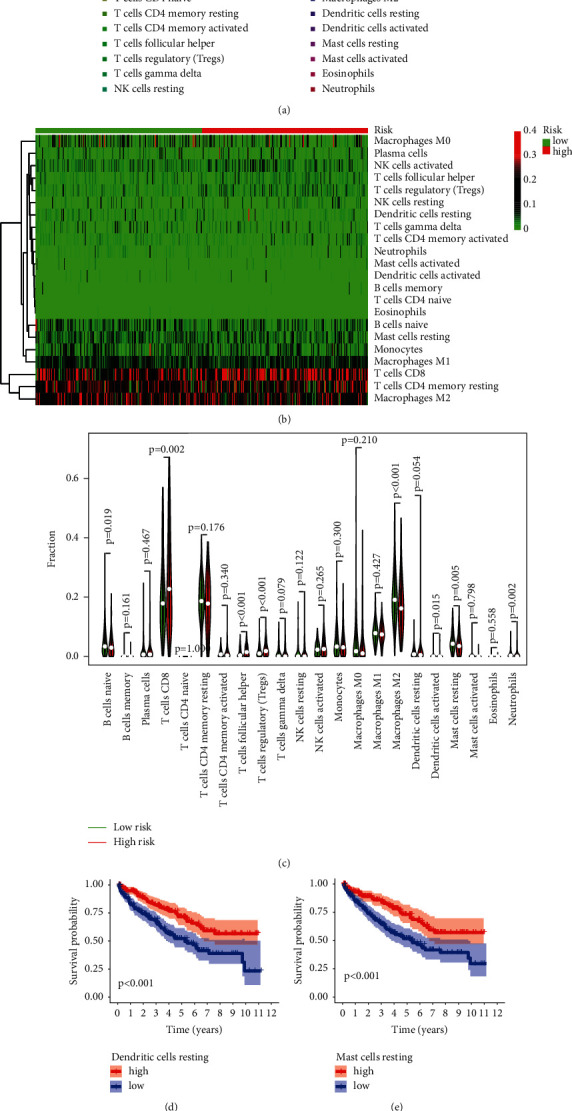
Composition of immune cells (a) and heat map (b) calculated by the CIBERSORT algorithm for each sample. (c) Differences in immune cell content between high- and low-risk groups. (d–g) Kaplan-Meier analysis of OS between high and low levels of the four immune cells.

We show the content of 22 immune cells in each sample by bar plot, as shown in Figure 7(a). Heat map of 22 immune cells is shown in Figure 7(b). Furthermore, using the Wilcoxon rank-sum test, we discovered that CD8T cells, naïve B cells, follicular helper T cells, regulatory T cells (Tregs), M2Macrophages, and resting mast cells were substantially different between high- and low-risk individuals (Figure 7(c)). Furthermore, K-M analysis indicated that patients with low proportions of resting dendritic and mast cells had a poor prognosis. Patients with a high number of follicular helper and regulatory T cells (Tregs) had a better prognosis (Figures 7(d)–7(g)).

### 3.8. GSEA

**Figure 8:**
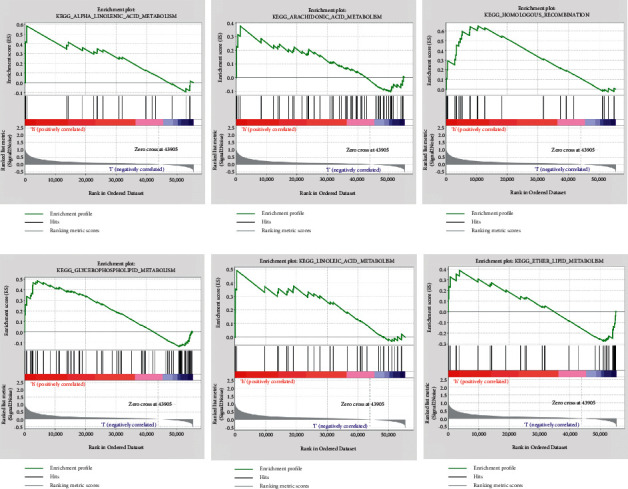
GSEA enrichment between the two groups.

We used GSEA analysis on the high- and low-risk groups to further study the essential signalling pathways. Our results showed that alpha linolenic acid metabolism, arachidonic acid metabolism, homologous recombination, glycerophospholipid metabolism, linoleic acid metabolism, and ether lipid metabolism were enriched (Figure 8).

### 3.9. Screening for DEGs

**Figure 9:**
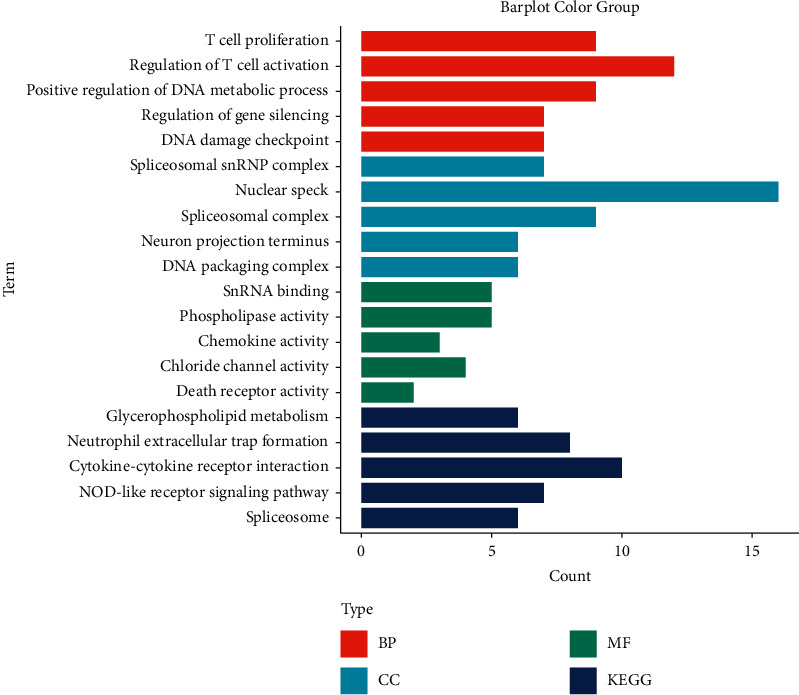
Pathway enrichment analysis for the DEGs.

We found 765 DEGs between high- and low-risk groups, comprising 6 downregulated and 759 upregulated genes (Figure S1). Based on these DEGs, we ran a pathway enrichment analysis. They were primarily enriched in immune-related pathways, such as T cell proliferation regulation and T cell activation (Figure 9).

### 3.10. Potential Small Molecule Drugs

**Figure 10:**
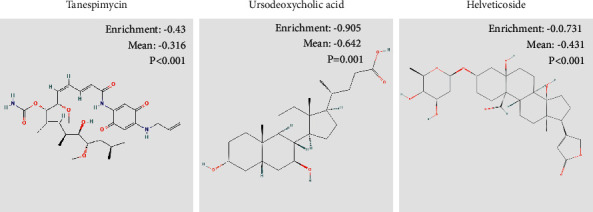
Two-dimensional diagram of the three most significant drugs.

We uploaded 765 DEGs with |log2FC| ≥1 to the CMap website. Among these highly correlated molecules, tanespimycin, ursodeoxycholic acid, and helveticoside had the highest degree of negative correlation with patients in the high-risk group of KIRC (Figure 10). All of them may have a potential therapeutic effect on KIRC patients in the high-risk group.

### 3.11. Crosstalk with Acute Kidney Injury

The incidence of tumors has increased and so has the number of patients with kidney injury caused by tumors. Acute kidney injury is a common complication of the cancer itself or the treatment process. To find the relationship between high- and low-risk groups of KIRC patients and AKI, we downloaded GSE139061, which contains 39 native human kidney biopsy samples (AKI group) and 9 reference nephrectomy samples (REF group); we found 44 shared DEGs (Figure S2). Future studies are needed to elucidate the relationship between KIRC and AKI.

## 4. Discussion

Kidney cancer has been on the rise in terms of incidence and death in recent years, and its treatment remains a serious issue globally, owing to its dismal prognosis [23]. As a result, identifying patients with a bad prognosis is a critical task. Despite the findings of various prognostic studies, TNM staging remains the most accurate predictor of KIRC [24]. However, because of the variability of KIRC, clinical outcomes among individuals with the same TNM stage may differ dramatically [25]. As a result, finding reliable prognostic biomarkers is critical for building an accurate prognosis model. Because of the role of the immune system in cancer growth and the existence of a unique immunological milieu in the kidney, the search for immune-related biomarkers is essential for the prognosis of KIRC patients and may help guide immunotherapy research.

We used IRG and IR-lncRNAs to create the first predictive model for KIRC patients in this research. We built and validated the predictive model based on seven IR-lncRNAs. The findings revealed that the predictive model correctly categorized KIRC patients into high- and low-risk groups, with substantial variations in OS between the risk groups. This predictive model's prognostic value was also verified, demonstrating that the predictive model based on IRG and IR-lncRNAs is reliable and valuable.

These seven IR-lncRNA indicators all showed a good correlation with prognosis. Other biomarkers utilized in our prognosis model have been reported by research, even though certain IR-lncRNAs in our predictive model have not been functionally annotated and elucidated. The lncRNA DLGAP1-AS1 has been demonstrated to increase Wnt1 transcription and gastric cancer growth by interacting with Six3 [26]. In breast cancer, DGUOK-AS1 may operate as a tumor promoter via modulating the miR-204-5p/IL-11 axis [27]. By modulating MMP14 expression, the lncRNA MELTF-AS1 may enhance osteosarcoma metastasis [28].

Immune cell infiltration of cancers has lately been associated with prognosis in many studies [29, 30]. The ESTIMATE algorithm analyzes the particular gene expression profile of immune cells and generates an immune score to forecast immune cell infiltration. Immune cell infiltration correlates with patient prognosis in many tumors, according to several prior ESTIMATE assessments [31]. We discovered that risk scores based on predictive models were favourably connected with immune scores in the current investigation. Patients with high immunological ratings also had a worse prognosis. To learn more about immune cell infiltration, we ran another CIBERSORT study to look at the different kinds of invading cells. CD8 T cells, regulatory T cells (Tregs), and follicular helper T cells were shown to be more prevalent in the high-risk group. Regulatory T cells (Tregs) and follicular helper T cells have also been linked to a poor prognosis. The follicular helper T cells may play a significant part in the progression of KIRC, which should be looked into more in the future. We found through GSEA analysis that alpha linolenic acid and arachidonic acid that maintain cell structure and function also enhance immune functions and regulate lipid metabolism and related gene expression [32, 33].

Despite attempts by researchers to enhance KIRC patients' prognosis, the prognosis of KIRC patients has remained bad for decades. In this investigation, three small compounds, tanespimycin, ursodeoxycholic acid, and helveticoside, were discovered to have potential therapeutic benefits in individuals with KIRC. Tanespimycin is a geldanamycin derivative that has been studied for use in the treatment of cancer, notably in young individuals with specific forms of leukemia or solid tumors [34, 35]. It is used to treat young kids with specific forms of leukemia or solid tumors, particularly kidney cancers. Helveticoside is a bioactive component of *Descurainia sophia* seed extract. Several investigations have shown this chemical to regulate genes involved in proliferation and apoptosis in caterpillar lung carcinoma cells [36]. Ursodeoxycholic acid is a secondary bile acid that is formed by intestinal bacteria and is necessary for lipid metabolism as well as the integrity of the intestinal barrier. Ursodeoxycholic acid has also been studied for its impact on tumor cell migration, tumor stem cells, and drug-induced dysbiosis [37, 38]. Ursodeoxycholic acid may also protect against the effects of cancer-fighting chemotherapeutics.

This study has some limitations. First, it is based on one public database. Second, there were no cohorts from other databases for validation. Therefore, a large multicenter study is needed to confirm our findings before our predictive model can be applied to the clinicians.

## 5. Conclusion

For the first time in KIRC patients, we identified and validated a predictive model consisting of 7 IR-lncRNAs with independent prognostic significance. In addition, our predictive model may provide a new basis for immunotherapy and immune targets for KIRC. In addition, based on this predictive model, we predicted three small molecule drugs with potential therapeutic value for KIRC treatment and identified genes that intersect with AKI.

## Figures and Tables

**Table 1:** Clinicopathological parameters of KIRC patients in this study.

Variables	Subgroups	*N*	%
Age	≤65	352	65.55
	>65	185	34.45

Gender	Male	346	64.43
	Female	191	35.57

Grade	G1	14	2.61
	G2	230	42.83
	G3	207	38.55
	G4	78	14.53
	Unknown	8	1.48

T classification	T1	275	51.21
	T2	69	12.85
	T3	182	33.89
	T4	11	2.05

N classification	N0	240	44.69
	N1	17	3.17
	Unknown	280	52.14

M classification	M0	426	79.33
	M1	79	14.71
	Unknown	32	5.96

UICC stage	Stage I	269	50.09
	Stage II	57	10.61
	Stage III	125	23.28
	Stage IV	83	15.46
	Unknown	3	0.56

Survival status	Alive	360	67.04
	Dead	177	32.96

**Table 2:** IR-lncRNAs from the predictive model of TCGA are associated with OS.

Gene	HR	95% CI	*P* value	LASSO coef.
DLGAP1-AS2	3.749	2.860–4.912	<0.001	0.140099926
AC024060.2	2.225	1.791–2.762	<0.001	0.041871909
SLBP-DT	2.628	2.057–3.355	<0.001	0.177728419
DGUOK-AS1	3.115	2.187–4.435	<0.001	0.085301062
MYG1-AS1	2.195	1.785–2.698	<0.001	0.141712499
AC005785.1	2.821	2.127–3.740	<0.001	0.164170337
MELTF-AS1	2.273	1.899–2.719	<0.001	0.192870454

**Table 3:** Correlation between the training cohort, validation cohort, and total cohort clinical features and the risk scores of the predictive model based on immune-related lncRNAs.

Variables	Training cohort (*n* = 264)				Validation cohort (*n* = 262)				Total cohort (*n* = 536)			
	High risk (%)	Low risk (%)	*χ* ^2^	*P*	High risk (%)	Low risk (%)	*χ* ^2^	*P*	High risk (%)	Low risk (%)	*χ* ^2^	*P*
Age			0.017	0.897			0	1			0.034	0.854
≤65	86 (65.15%)	88 (66.67%)			86 (65.65%)	87 (66.41%)			172 (65.4%)	175 (66.54%)		
>65	46 (34.85%)	44 (33.33%)			45 (34.35%)	44 (33.59%)			91 (34.6%)	88 (33.46%)		
Gender			0	1			0	1			0	1
Female	44 (33.33%)	44 (33.33%)			47 (35.88%)	48 (36.64%)			91 (34.6%)	92 (34.98%)		
Male	88 (66.67%)	88 (66.67%)			84 (64.12%)	83 (63.36%)			172 (65.4%)	171 (65.02%)		
Grade			0.962	0.327			1.880	0.170			3.056	0.08
G1-2	54 (40.91%)	62 (46.97%)			56 (42.75%)	67 (51.15%)			110 (41.83%)	129 (49.05%)		
G3-4	78 (59.09%)	68 (51.52%)			73 (55.73%)	60 (45.8%)			151 (57.41%)	128 (48.67%)		
Unknown	0 (0%)	2 (1.52%)			2 (1.53%)	4 (3.05%)			2 (0.76%)	6 (2.28%)		
Stage			8.339	0.004			0.666	0.414			7.41	0.006
Stages I-II	67 (50.76%)	91 (68.94%)			76 (58.02%)	84 (64.12%)			143 (54.37%)	175 (66.54%)		
Stages III-IV	65 (49.24%)	41 (31.06%)			53 (40.46%)	46 (35.11%)			118 (44.87%)	87 (33.08%)		
Unknown	/	/			2 (1.53%)	1 (0.76%)			2 (0.76%)	1 (0.38%)		
T classification			5.196	0.023			2.450	0.117			7.918	0.005
T1-2	72 (54.55%)	91 (68.94%)			80 (61.07%)	93 (70.99%)			152 (57.79%)	184 (69.96%)		
T3-4	60 (45.45%)	41 (31.06%)			51 (38.93%)	38 (29.01%)			111 (42.21%)	79 (30.04%)		
M classification			11.324	<0.001			0	1			5.99	0.014
M0	94 (71.21%)	119 (90.15%)			97 (74.05%)	108 (82.44%)			191 (72.62%)	227 (86.31%)		
M1	28 (21.21%)	9 (6.82%)			20 (15.27%)	21 (16.03%)			48 (18.25%)	30 (11.41%)		
Unknown	10 (7.58%)	4 (3.03%)			14 (10.69%)	2 (1.53%)			24 (9.13%)	6 (2.28%)		
N classification			1.292	0.256			0.145	0.703			0.016	0.898
N0	61 (46.21%)	59 (44.7%)			61 (46.56%)	57 (43.51%)			122 (46.39%)	116 (44.11%)		
N1	5 (3.79%)	1 (0.76%)			4 (3.05%)	6 (4.58%)			9 (3.42%)	7 (2.66%)		
Unknown	66 (50%)	72 (54.55%)			66 (50.38%)	68 (51.91%)			132 (50.19%)	140 (53.23%)		

## Data Availability

Publicly available datasets were analyzed in this study, and they are available from the Gene Expression Omnibus (GEO) and in The Cancer Genome Atlas (TCGA) database.

## Supplementary Materials

Supplementary Materials Table S1: 2683 IRGs from ImmPort Shared Data. Table S2 : IRGs in the red module. Table S3 : IRGs in the grey module. Table S4: 63 prognostic IRGs. Table S5 : 206 prognostic IR-lncRNAs. Figure S1 : volcano plot showing 765 DEGs between high- and low-risk groups. Figure S2: 44 shared DEGs between KIRC and AKI.
