# Supplementary material for: Exploration of Prognostic Immune-Related Genes and lncRNAs Biomarkers in Kidney Renal Clear Cell Carcinoma and Its Crosstalk with Acute Kidney Injury
Source: J Oncol. 2022 Feb 8;2022:6100187. doi: 10.1155/2022/6100187 (PMC8847043; doi:10.1155/2022/6100187)
Supplement: Supplementary Materials — Table S1: 2683 IRGs from ImmPort Shared Data. Table S2 : IRGs in the red module. Table S3 : IRGs in the grey module. Table S4: 63 prognostic IRGs. Table S5 : 206 prognostic IR-lncRNAs. Figure S1 : volcano plot showing 765 DEGs between high- and low-risk groups. Figure S2: 44 shared DEGs between KIRC and AKI. [file 6100187.f1.zip › 6100187.f1/Figure S2.pdf]

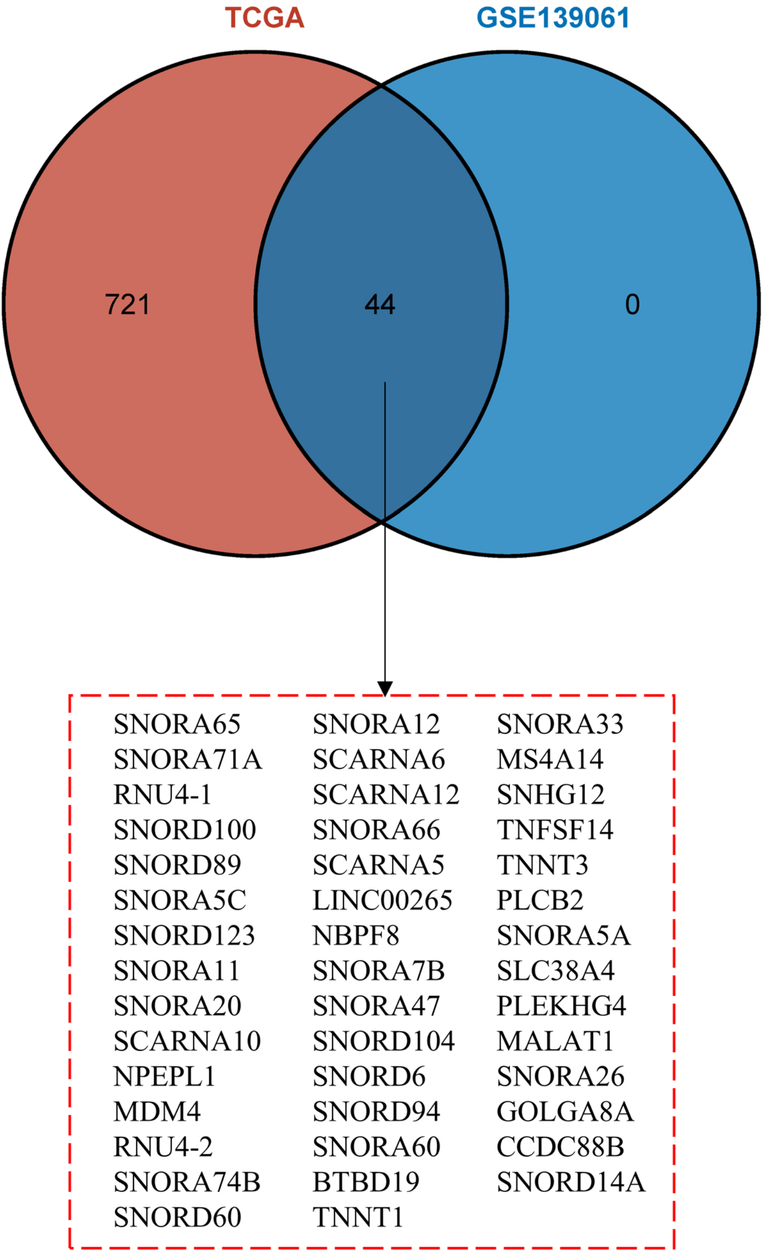
TCGA
GSE139061
721
44
0
SNORA65
SNORA71A
RNU4-1
SNORD100
SNORD89
SNORA5C
SNORD123
SNORA11
SNORA20
SCARNA10
NPEPL1
MDM4
RNU4-2
SNORA74B
SNORD60
SNORA12
SCARNA6
SCARNA12
SNORA66
SCARNA5
LINC00265
NBPF8
SNORA7B
SNORA47
SNORD104
SNORD6
SNORD94
SNORA60
BTBD19
TNNT1
SNORA33
MS4A14
SNHG12
TNFSF14
TNNT3
PLCB2
SNORA5A
SLC38A4
PLEKHG4
MALAT1
SNORA26
GOLGA8A
CCDC88B
SNORD14A
