# Supplementary material for: Exploration of Prognostic Immune-Related Genes and lncRNAs Biomarkers in Kidney Renal Clear Cell Carcinoma and Its Crosstalk with Acute Kidney Injury
Source: J Oncol. 2022 Feb 8;2022:6100187. doi: 10.1155/2022/6100187 (PMC8847043; doi:10.1155/2022/6100187)
Supplement: Supplementary Materials — Table S1: 2683 IRGs from ImmPort Shared Data. Table S2 : IRGs in the red module. Table S3 : IRGs in the grey module. Table S4: 63 prognostic IRGs. Table S5 : 206 prognostic IR-lncRNAs. Figure S1 : volcano plot showing 765 DEGs between high- and low-risk groups. Figure S2: 44 shared DEGs between KIRC and AKI. [file 6100187.f1.zip › 6100187.f1/Table S2.docx]

Table S2. IRGs in red module

CTSL

IFNA1

IFNA5

MICA

NFYC

IFI30

CXCL2

CXCL3

LCN12

LMBR1L

DEFB124

WFIKKN1

IRF3

OBP2B

FABP12

MUC4

KLKB1

TYK2

NFKBIZ

ROBO3

SLC11A1

SKIV2L

TNFRSF10B

LTB4R

MASP2

TRIM27

DDX17

IRF9

AGER

ACO1

CCL16

IL4

CHP1

IKBKB

AKT2

GPR17

LTB4R2

PLXNA3

PLXNB1

AMH

CDNF

CGB3

CLCF1

CORT

FGF17

GDF9

GNRH1

GNRH2

GPHA2

IFNE

IL9

INSL5

LHB

LRSAM1

MSTN

NODAL

UCN

VIP

BRD8

EPOR

ESR2

GIPR

IL11RA

IL18RAP

MC1R

NR2C1

RXRB

SCTR

TNFRSF14

TNFRSF25

LAT

SH3BP2

SHC2

MAP3K8
