# Supplementary material for: Exploration of Prognostic Immune-Related Genes and lncRNAs Biomarkers in Kidney Renal Clear Cell Carcinoma and Its Crosstalk with Acute Kidney Injury
Source: J Oncol. 2022 Feb 8;2022:6100187. doi: 10.1155/2022/6100187 (PMC8847043; doi:10.1155/2022/6100187)
Supplement: Supplementary Materials — Table S1: 2683 IRGs from ImmPort Shared Data. Table S2 : IRGs in the red module. Table S3 : IRGs in the grey module. Table S4: 63 prognostic IRGs. Table S5 : 206 prognostic IR-lncRNAs. Figure S1 : volcano plot showing 765 DEGs between high- and low-risk groups. Figure S2: 44 shared DEGs between KIRC and AKI. [file 6100187.f1.zip › 6100187.f1/Table S3.docx]

Table S3. IRGs in grey module

IFNA2

IFNA4

IFNA7

IFNA14

IFNA17

PDIA2

DEFB4A

CXCL6

DEFB103B

DEFB103A

DEFA6

DEFA5

LCN1

COLEC10

DEFB106A

PENK

LCN9

S100A7

DEFB104A

DEFB104B

S100A7A

TMSB4Y

DEFB130A

DEFB114

DEFB112

DEFB4B

S100G

DEFB130B

PF4V1

MBL2

RBP4

NOX4

FABP2

FABP9

LCNL1

PI15

AEN

ADIPOQ

SEMG1

PGC

SEMG2

PROC

GFAP

CCL27

CRP

C5

FGF10

LECT2

PTGDR2

BMP10

BMP15

CTF1

FAM3D

FGF11

GKN1

GRP

IAPP

IL17F

IL3

INS

NDP

NPY

PRL

PROK1

PTH

RLN1

SST

TAC1

TSLP

AGTR2

CNTFR

GHSR

GLP1R

GLP2R

IL13RA2

LGR5

MC3R

NR2E1

TNFRSF10C

NCR2
