# Supplementary material for: Exploration of Prognostic Immune-Related Genes and lncRNAs Biomarkers in Kidney Renal Clear Cell Carcinoma and Its Crosstalk with Acute Kidney Injury
Source: J Oncol. 2022 Feb 8;2022:6100187. doi: 10.1155/2022/6100187 (PMC8847043; doi:10.1155/2022/6100187)
Supplement: Supplementary Materials — Table S1: 2683 IRGs from ImmPort Shared Data. Table S2 : IRGs in the red module. Table S3 : IRGs in the grey module. Table S4: 63 prognostic IRGs. Table S5 : 206 prognostic IR-lncRNAs. Figure S1 : volcano plot showing 765 DEGs between high- and low-risk groups. Figure S2: 44 shared DEGs between KIRC and AKI. [file 6100187.f1.zip › 6100187.f1/Table S4.docx]

Table S4. 63 prognostic IRGs

| gene | HR | HR.95L | HR.95H | pvalue |
| --- | --- | --- | --- | --- |
| PDIA2 | 1.361248 | 1.123445 | 1.649387 | 0.001643 |
| DEFB4A | 2.167935 | 1.184751 | 3.967028 | 0.012077 |
| CXCL6 | 1.174416 | 1.064593 | 1.295568 | 0.00133 |
| S100A7 | 2.02776 | 1.443776 | 2.847957 | 4.52E-05 |
| TMSB4Y | 0.730841 | 0.546058 | 0.978153 | 0.034988 |
| PF4V1 | 1.175522 | 1.031739 | 1.339342 | 0.015126 |
| NOX4 | 0.778657 | 0.65087 | 0.931533 | 0.00623 |
| LCNL1 | 1.951459 | 1.254581 | 3.035431 | 0.003015 |
| PI15 | 1.888088 | 1.435543 | 2.483294 | 5.47E-06 |
| ADIPOQ | 1.459869 | 1.097453 | 1.941965 | 0.009358 |
| CRP | 1.208418 | 1.097153 | 1.330966 | 0.000122 |
| PTGDR2 | 0.159514 | 0.038849 | 0.654974 | 0.010861 |
| FGF11 | 1.331956 | 1.046186 | 1.695786 | 0.019997 |
| GRP | 1.313528 | 1.027237 | 1.679609 | 0.02969 |
| PROK1 | 14.78862 | 2.37525 | 92.07588 | 0.003888 |
| PTH | 8.999958 | 2.734567 | 29.6205 | 0.0003 |
| TSLP | 2.79742 | 1.525246 | 5.130684 | 0.000887 |
| CTSL | 0.709556 | 0.533066 | 0.944478 | 0.018701 |
| IFI30 | 1.963513 | 1.578648 | 2.442207 | 1.35E-09 |
| CXCL2 | 1.284311 | 1.15847 | 1.423822 | 1.98E-06 |
| CXCL3 | 1.659492 | 1.336581 | 2.060416 | 4.48E-06 |
| LMBR1L | 1.824035 | 1.406673 | 2.365229 | 5.79E-06 |
| DEFB124 | 7.769322 | 2.616144 | 23.07303 | 0.000223 |
| WFIKKN1 | 1.483034 | 1.17629 | 1.869769 | 0.000858 |
| IRF3 | 2.370453 | 1.772626 | 3.169902 | 5.86E-09 |
| OBP2B | 4.385574 | 1.127539 | 17.05773 | 0.03291 |
| FABP12 | 1521.021 | 57.609 | 40158.72 | 1.15E-05 |
| TYK2 | 2.034488 | 1.380827 | 2.997583 | 0.000328 |
| NFKBIZ | 1.529855 | 1.327219 | 1.763429 | 4.49E-09 |
| ROBO3 | 2.004094 | 1.579475 | 2.542865 | 1.05E-08 |
| SLC11A1 | 1.832492 | 1.506408 | 2.229162 | 1.38E-09 |
| SKIV2L | 2.305271 | 1.533473 | 3.465516 | 5.93E-05 |
| TNFRSF10B | 1.464852 | 1.130663 | 1.897817 | 0.003859 |
| LTB4R | 1.867835 | 1.530806 | 2.279065 | 7.56E-10 |
| TRIM27 | 2.631904 | 1.537127 | 4.506406 | 0.000421 |
| IRF9 | 1.686233 | 1.370742 | 2.074337 | 7.66E-07 |
| AGER | 1.55063 | 1.308444 | 1.837643 | 4.13E-07 |
| ACO1 | 0.571127 | 0.427941 | 0.762222 | 0.000143 |
| IL4 | 106.019 | 33.3458 | 337.0745 | 2.74E-15 |
| CHP1 | 0.589867 | 0.412091 | 0.844335 | 0.003919 |
| LTB4R2 | 2.031575 | 1.454468 | 2.837669 | 3.22E-05 |
| PLXNA3 | 1.608658 | 1.244259 | 2.079778 | 0.000286 |
| AMH | 2.473961 | 1.988805 | 3.077467 | 4.18E-16 |
| CORT | 2.996624 | 1.592328 | 5.63939 | 0.000669 |
| FGF17 | 7.08842 | 3.088438 | 16.26897 | 3.83E-06 |
| GDF9 | 4.046505 | 1.825566 | 8.969386 | 0.000577 |
| GNRH1 | 1.891565 | 1.513423 | 2.36419 | 2.13E-08 |
| GNRH2 | 6.365813 | 2.238267 | 18.10489 | 0.000519 |
| IFNE | 1.772021 | 1.401201 | 2.240976 | 1.79E-06 |
| INSL5 | 6.830183 | 2.590359 | 18.00963 | 0.000103 |
| LHB | 5.026308 | 3.225137 | 7.833394 | 9.86E-13 |
| MSTN | 1.700323 | 1.114514 | 2.594044 | 0.013777 |
| NODAL | 3.583307 | 1.209166 | 10.61896 | 0.0213 |
| UCN | 2.16624 | 1.748515 | 2.683761 | 1.53E-12 |
| EPOR | 1.657585 | 1.32788 | 2.069153 | 7.96E-06 |
| ESR2 | 2.105201 | 1.198462 | 3.697968 | 0.009603 |
| GIPR | 2.535174 | 1.790664 | 3.589233 | 1.57E-07 |
| IL11RA | 1.387958 | 1.042608 | 1.847702 | 0.024717 |
| MC1R | 2.445874 | 1.807045 | 3.310542 | 7.00E-09 |
| NR2C1 | 2.41127 | 1.639845 | 3.545594 | 7.67E-06 |
| TNFRSF25 | 1.494333 | 1.241115 | 1.799215 | 2.23E-05 |
| LAT | 2.629755 | 1.787907 | 3.867994 | 9.04E-07 |
| MAP3K8 | 1.887316 | 1.483062 | 2.401762 | 2.41E-07 |
