# Supplementary material for: Exploration of Prognostic Immune-Related Genes and lncRNAs Biomarkers in Kidney Renal Clear Cell Carcinoma and Its Crosstalk with Acute Kidney Injury
Source: J Oncol. 2022 Feb 8;2022:6100187. doi: 10.1155/2022/6100187 (PMC8847043; doi:10.1155/2022/6100187)
Supplement: Supplementary Materials — Table S1: 2683 IRGs from ImmPort Shared Data. Table S2 : IRGs in the red module. Table S3 : IRGs in the grey module. Table S4: 63 prognostic IRGs. Table S5 : 206 prognostic IR-lncRNAs. Figure S1 : volcano plot showing 765 DEGs between high- and low-risk groups. Figure S2: 44 shared DEGs between KIRC and AKI. [file 6100187.f1.zip › 6100187.f1/Table S5.docx]

Table S5. 206 prognostic IR-lncRNAs

| gene | HR | HR.95L | HR.95H | pvalue |
| --- | --- | --- | --- | --- |
| AC110285.2 | 1.37273 | 1.171963 | 1.60789 | 8.61E-05 |
| AC010973.2 | 2.292729 | 1.7809 | 2.951658 | 1.21E-10 |
| AC007038.1 | 1.772983 | 1.4322 | 2.194853 | 1.45E-07 |
| AC092119.2 | 2.350254 | 1.771822 | 3.117521 | 3.06E-09 |
| AC093788.1 | 2.549845 | 1.898419 | 3.424803 | 5.01E-10 |
| AC232271.1 | 2.004909 | 1.537721 | 2.614037 | 2.76E-07 |
| AC005840.4 | 1.695738 | 1.284598 | 2.238465 | 0.000193 |
| AC008760.1 | 1.719197 | 1.298038 | 2.277004 | 0.000157 |
| ARHGAP27P1-BPTFP1-KPNA2P3 | 1.589732 | 1.265741 | 1.996654 | 6.70E-05 |
| AL021707.8 | 1.53424 | 1.239764 | 1.898663 | 8.27E-05 |
| AL354836.1 | 1.414124 | 1.215809 | 1.644787 | 6.97E-06 |
| AC018653.3 | 1.83233 | 1.406174 | 2.387636 | 7.33E-06 |
| AC092171.4 | 1.947789 | 1.531585 | 2.477095 | 5.46E-08 |
| AC087741.1 | 1.72592 | 1.396326 | 2.133313 | 4.47E-07 |
| DLGAP1-AS2 | 3.748525 | 2.860478 | 4.912269 | 9.83E-22 |
| NALT1 | 2.106748 | 1.650065 | 2.689826 | 2.27E-09 |
| AC074117.1 | 2.775934 | 1.944172 | 3.963545 | 1.92E-08 |
| AC002553.2 | 1.87108 | 1.429206 | 2.449571 | 5.16E-06 |
| AL450384.2 | 1.519844 | 1.236261 | 1.868477 | 7.10E-05 |
| AC127502.2 | 1.826098 | 1.470091 | 2.268318 | 5.25E-08 |
| STAG3L5P-PVRIG2P-PILRB | 1.636574 | 1.292584 | 2.072107 | 4.28E-05 |
| AC003102.1 | 1.882376 | 1.487753 | 2.381671 | 1.37E-07 |
| AC012615.6 | 1.865862 | 1.404784 | 2.478276 | 1.66E-05 |
| AC010245.2 | 2.829394 | 1.884222 | 4.248688 | 5.33E-07 |
| AC024060.2 | 2.224727 | 1.791476 | 2.762755 | 4.62E-13 |
| SLBP-DT | 2.627578 | 2.057537 | 3.355548 | 9.75E-15 |
| AC008610.1 | 1.696072 | 1.4215 | 2.023679 | 4.54E-09 |
| AL031670.1 | 2.150341 | 1.551167 | 2.98096 | 4.34E-06 |
| AC104564.3 | 1.789106 | 1.383284 | 2.313987 | 9.34E-06 |
| AC016957.2 | 2.350659 | 1.60209 | 3.448994 | 1.25E-05 |
| DGUOK-AS1 | 3.114838 | 2.187568 | 4.435159 | 2.95E-10 |
| ANKRD10-IT1 | 1.380334 | 1.162489 | 1.639003 | 2.35E-04 |
| AC095057.3 | 3.270313 | 2.330702 | 4.588723 | 7.07E-12 |
| BACE1-AS | 1.992411 | 1.444646 | 2.747871 | 2.64E-05 |
| VPS9D1-AS1 | 2.321943 | 1.802934 | 2.990361 | 6.74E-11 |
| BCRP3 | 1.579456 | 1.268813 | 1.966153 | 4.30E-05 |
| AL135999.1 | 1.830147 | 1.418652 | 2.361001 | 3.30E-06 |
| SCAT2 | 2.311969 | 1.856605 | 2.879021 | 6.96E-14 |
| AL683807.1 | 1.823788 | 1.423922 | 2.335943 | 1.95E-06 |
| AC008735.2 | 1.400834 | 1.201533 | 1.633194 | 1.67E-05 |
| AC005899.6 | 1.539273 | 1.23572 | 1.917395 | 1.19E-04 |
| THUMPD3-AS1 | 2.196882 | 1.585533 | 3.043957 | 2.24E-06 |
| DM1-AS | 2.177421 | 1.520927 | 3.117285 | 2.13E-05 |
| AL031186.1 | 2.08954 | 1.517055 | 2.878062 | 6.44E-06 |
| AC010761.1 | 1.894748 | 1.488717 | 2.411518 | 2.06E-07 |
| AC010326.3 | 1.880838 | 1.508393 | 2.345246 | 2.01E-08 |
| MYG1-AS1 | 2.195219 | 1.785497 | 2.698961 | 8.66E-14 |
| CD27-AS1 | 2.657383 | 1.977798 | 3.570478 | 8.84E-11 |
| LENG8-AS1 | 1.526715 | 1.258193 | 1.852544 | 1.81E-05 |
| AC063948.1 | 2.568258 | 1.895528 | 3.479742 | 1.15E-09 |
| CCDC18-AS1 | 1.67095 | 1.367 | 2.042483 | 5.39E-07 |
| PRANCR | 2.38141 | 1.801393 | 3.148183 | 1.11E-09 |
| AL117209.1 | 1.647714 | 1.225751 | 2.214936 | 0.000938 |
| NDUFV2-AS1 | 2.167445 | 1.408395 | 3.335583 | 4.37E-04 |
| LINC01089 | 1.742596 | 1.423876 | 2.132658 | 7.09E-08 |
| LAMC1-AS1 | 1.906529 | 1.333102 | 2.726613 | 0.000408 |
| AC132192.2 | 2.200587 | 1.696187 | 2.854984 | 2.89E-09 |
| AL008582.1 | 1.786347 | 1.388818 | 2.297663 | 6.26E-06 |
| AC093726.2 | 2.422688 | 1.756866 | 3.340846 | 6.77E-08 |
| AP001160.1 | 2.31061 | 1.771526 | 3.013742 | 6.46E-10 |
| AC090589.3 | 1.67317 | 1.29503 | 2.161725 | 8.22E-05 |
| SNHG10 | 2.028359 | 1.537565 | 2.675817 | 5.63E-07 |
| HOTAIRM1 | 1.885778 | 1.580209 | 2.250436 | 2.02E-12 |
| ARRDC1-AS1 | 1.816889 | 1.362055 | 2.423607 | 4.87E-05 |
| MCCC1-AS1 | 2.295988 | 1.704807 | 3.092175 | 4.45E-08 |
| LINC01176 | 1.392071 | 1.158298 | 1.673025 | 0.000421 |
| AC073487.1 | 2.067099 | 1.497598 | 2.853167 | 1.01E-05 |
| AC253536.6 | 1.883814 | 1.404298 | 2.527066 | 2.39E-05 |
| ZNF32-AS2 | 1.877355 | 1.375035 | 2.563179 | 7.35E-05 |
| AC129510.1 | 1.992375 | 1.561888 | 2.541511 | 2.86E-08 |
| AL157392.4 | 1.985896 | 1.457124 | 2.706552 | 1.40E-05 |
| RASGRP3-AS1 | 2.601829 | 1.949299 | 3.472796 | 8.55E-11 |
| AC009065.8 | 1.648107 | 1.290906 | 2.104148 | 6.11E-05 |
| AC048382.2 | 1.900506 | 1.395531 | 2.588206 | 4.60E-05 |
| AC010883.1 | 1.637059 | 1.315167 | 2.037736 | 1.02E-05 |
| ASB16-AS1 | 2.515429 | 1.793398 | 3.528153 | 9.10E-08 |
| AC048341.2 | 1.656894 | 1.418635 | 1.935168 | 1.83E-10 |
| AC105020.1 | 1.592799 | 1.300663 | 1.950552 | 6.71E-06 |
| AC010542.5 | 1.720266 | 1.382285 | 2.140886 | 1.17E-06 |
| AC084824.5 | 2.19887 | 1.689694 | 2.861483 | 4.54E-09 |
| LINC01138 | 2.437523 | 1.807656 | 3.286863 | 5.18E-09 |
| AC004253.1 | 1.861195 | 1.416742 | 2.445079 | 8.11E-06 |
| PTOV1-AS1 | 3.42396 | 2.16312 | 5.419717 | 1.50E-07 |
| AL359504.1 | 2.251973 | 1.651965 | 3.069908 | 2.82E-07 |
| AC011472.1 | 1.386751 | 1.154568 | 1.665626 | 0.00047 |
| AP000254.2 | 2.083252 | 1.545701 | 2.807748 | 1.44E-06 |
| AL136295.2 | 1.593453 | 1.218235 | 2.084239 | 0.000672 |
| AC011498.6 | 1.929954 | 1.38043 | 2.698236 | 0.00012 |
| AC093484.4 | 1.826713 | 1.344218 | 2.482397 | 0.000118 |
| AC008105.2 | 1.821626 | 1.475767 | 2.248542 | 2.37E-08 |
| SH3BP5-AS1 | 1.501614 | 1.19188 | 1.891839 | 0.000562 |
| AC092794.1 | 2.25388 | 1.558177 | 3.260205 | 1.60E-05 |
| AP001767.2 | 2.077151 | 1.472123 | 2.930839 | 3.16E-05 |
| AL390728.5 | 1.61517 | 1.346379 | 1.937623 | 2.44E-07 |
| PDXDC2P-NPIPB14P | 2.188207 | 1.61535 | 2.964219 | 4.27E-07 |
| AC005785.1 | 2.821013 | 2.127686 | 3.740268 | 5.74E-13 |
| AL022322.1 | 1.430564 | 1.178971 | 1.735846 | 0.000285 |
| DICER1-AS1 | 1.590459 | 1.237849 | 2.043512 | 0.000285 |
| AL513320.1 | 1.689604 | 1.36725 | 2.087959 | 1.20E-06 |
| AC010201.2 | 1.569913 | 1.215725 | 2.02729 | 0.000545 |
| AC020558.2 | 2.411275 | 1.748603 | 3.325081 | 7.94E-08 |
| AC009120.2 | 1.699901 | 1.35866 | 2.126849 | 3.47E-06 |
| MHENCR | 1.581897 | 1.333214 | 1.876966 | 1.47E-07 |
| AC005332.5 | 2.195709 | 1.601688 | 3.010037 | 1.02E-06 |
| AC069281.2 | 2.895931 | 2.076203 | 4.039306 | 3.78E-10 |
| LINC00106 | 1.297526 | 1.125741 | 1.495526 | 0.000325 |
| AC048341.1 | 2.466433 | 1.776169 | 3.42495 | 7.07E-08 |
| LMNTD2-AS1 | 1.269525 | 1.128081 | 1.428704 | 7.51E-05 |
| MELTF-AS1 | 2.272574 | 1.899373 | 2.719103 | 2.99E-19 |
| LINC00653 | 2.218257 | 1.722019 | 2.857497 | 6.98E-10 |
| AP006621.3 | 1.454292 | 1.167815 | 1.811045 | 0.00082 |
| AC012615.1 | 2.041866 | 1.543396 | 2.701325 | 5.76E-07 |
| AC002553.1 | 1.797919 | 1.391603 | 2.32287 | 7.18E-06 |
| FAM13A-AS1 | 1.655527 | 1.298623 | 2.110521 | 4.72E-05 |
| AC009283.1 | 1.681161 | 1.332587 | 2.120914 | 1.18E-05 |
| LINC00926 | 2.55045 | 1.852291 | 3.511758 | 9.62E-09 |
| AC244197.2 | 1.749563 | 1.408274 | 2.17356 | 4.37E-07 |
| CR936218.1 | 1.579628 | 1.217692 | 2.049143 | 0.000575 |
| SUGT1-DT | 2.142687 | 1.616458 | 2.840229 | 1.16E-07 |
| PVT1 | 1.864033 | 1.476652 | 2.353038 | 1.61E-07 |
| AC025171.4 | 1.816111 | 1.495725 | 2.205122 | 1.68E-09 |
| AC034236.2 | 2.022349 | 1.511928 | 2.705086 | 2.08E-06 |
| ITGB2-AS1 | 1.48839 | 1.252612 | 1.768549 | 6.20E-06 |
| AC245884.8 | 1.601934 | 1.326498 | 1.934563 | 9.82E-07 |
| HOXB-AS1 | 1.587337 | 1.284782 | 1.961141 | 1.85E-05 |
| AL391684.1 | 2.107735 | 1.497874 | 2.965903 | 1.88E-05 |
| AC010168.2 | 1.811896 | 1.34446 | 2.441847 | 9.45E-05 |
| AC116914.2 | 1.553607 | 1.314979 | 1.835539 | 2.24E-07 |
| AC106782.5 | 1.846981 | 1.311016 | 2.602058 | 0.000451 |
| AC004908.3 | 2.363923 | 1.805543 | 3.094986 | 3.91E-10 |
| LINC02604 | 1.771645 | 1.47671 | 2.125485 | 7.47E-10 |
| AC004148.1 | 1.682315 | 1.368178 | 2.068579 | 8.12E-07 |
| AC011462.4 | 1.679635 | 1.398001 | 2.018005 | 3.06E-08 |
| LINC00342 | 1.603787 | 1.34194 | 1.916726 | 2.06E-07 |
| TPT1-AS1 | 1.790326 | 1.280629 | 2.502887 | 0.000657 |
| ZNF460-AS1 | 1.874097 | 1.394572 | 2.518506 | 3.10E-05 |
| AL928654.2 | 1.39794 | 1.167518 | 1.673837 | 0.000267 |
| AC138207.4 | 1.719502 | 1.286592 | 2.298075 | 0.000249 |
| AL022328.3 | 2.619362 | 1.806748 | 3.79746 | 3.74E-07 |
| AC127024.5 | 1.79094 | 1.386933 | 2.312632 | 7.91E-06 |
| AC005840.2 | 1.919176 | 1.472736 | 2.500949 | 1.40E-06 |
| AP002807.1 | 2.253789 | 1.76894 | 2.871532 | 4.86E-11 |
| AC002128.1 | 1.715235 | 1.256097 | 2.342202 | 0.000688 |
| MINCR | 2.16521 | 1.666588 | 2.813013 | 7.26E-09 |
| N4BP2L2-IT2 | 1.90341 | 1.439064 | 2.517587 | 6.45E-06 |
| AC020907.4 | 1.983329 | 1.597826 | 2.461842 | 5.31E-10 |
| INE1 | 1.799945 | 1.386893 | 2.336016 | 9.92E-06 |
| THBS3-AS1 | 2.513582 | 1.793378 | 3.523013 | 8.75E-08 |
| AC026471.4 | 1.639937 | 1.364047 | 1.971629 | 1.41E-07 |
| AC021078.1 | 1.533601 | 1.238788 | 1.898574 | 8.64E-05 |
| AC127024.4 | 1.477623 | 1.19965 | 1.820005 | 0.000241 |
| MMP25-AS1 | 1.581641 | 1.252887 | 1.99666 | 0.000115 |
| AL031600.1 | 1.65752 | 1.283359 | 2.140768 | 0.000108 |
| AL355488.1 | 1.818297 | 1.474496 | 2.242261 | 2.25E-08 |
| AP006621.2 | 1.428045 | 1.203612 | 1.694327 | 4.42E-05 |
| Z69706.1 | 1.730138 | 1.306681 | 2.290825 | 0.000129 |
| AL136295.7 | 1.753198 | 1.304875 | 2.355555 | 0.000195 |
| AC015813.1 | 1.611004 | 1.319313 | 1.967185 | 2.88E-06 |
| AC011481.1 | 1.573842 | 1.296742 | 1.910155 | 4.44E-06 |
| AC018638.7 | 1.807081 | 1.345322 | 2.427331 | 8.49E-05 |
| GARS1-DT | 1.857079 | 1.429721 | 2.412177 | 3.50E-06 |
| AC108673.3 | 1.764271 | 1.461047 | 2.130426 | 3.62E-09 |
| AC027796.4 | 1.919332 | 1.560102 | 2.361277 | 6.98E-10 |
| AC005253.1 | 1.775819 | 1.296738 | 2.431896 | 0.000344 |
| ASMTL-AS1 | 1.402871 | 1.233152 | 1.595949 | 2.67E-07 |
| AL031714.1 | 1.913291 | 1.402063 | 2.610925 | 4.30E-05 |
| AC004687.1 | 1.442966 | 1.17014 | 1.779404 | 0.000605 |
| LINC01011 | 1.993445 | 1.394778 | 2.849072 | 0.000153 |
| LINC01004 | 1.905367 | 1.555011 | 2.334661 | 5.02E-10 |
| AC006435.2 | 1.719228 | 1.352071 | 2.186088 | 9.83E-06 |
| AC084018.1 | 1.403466 | 1.185525 | 1.661472 | 8.27E-05 |
| AC090948.3 | 1.990987 | 1.416195 | 2.79907 | 7.43E-05 |
| PSPC1-AS2 | 2.324137 | 1.695737 | 3.185407 | 1.58E-07 |
| AP003352.1 | 2.058565 | 1.613787 | 2.625928 | 6.12E-09 |
| LINC00115 | 2.288381 | 1.689624 | 3.099322 | 8.85E-08 |
| HM13-IT1 | 1.868276 | 1.476727 | 2.363641 | 1.90E-07 |
| AC132872.1 | 1.738754 | 1.377778 | 2.194306 | 3.17E-06 |
| ZNF436-AS1 | 2.106462 | 1.5916 | 2.787875 | 1.89E-07 |
| AC005104.1 | 1.747645 | 1.377718 | 2.2169 | 4.21E-06 |
| AC009118.3 | 1.952907 | 1.338463 | 2.849421 | 0.000516 |
| ZKSCAN2-DT | 2.173241 | 1.648134 | 2.86565 | 3.78E-08 |
| AL049780.1 | 1.679945 | 1.273755 | 2.215667 | 0.000239 |
| AL136304.1 | 1.955938 | 1.420437 | 2.693321 | 3.95E-05 |
| PTOV1-AS2 | 1.496081 | 1.26252 | 1.772849 | 3.29E-06 |
| AC003070.1 | 1.498767 | 1.196608 | 1.877224 | 0.000428 |
| U62317.1 | 1.642455 | 1.397729 | 1.930029 | 1.66E-09 |
| LINC00893 | 1.779177 | 1.366891 | 2.315818 | 1.84E-05 |
| AL162586.1 | 1.761479 | 1.39102 | 2.2306 | 2.61E-06 |
| KMT2E-AS1 | 1.452768 | 1.206901 | 1.748723 | 7.89E-05 |
| LINC01355 | 2.057284 | 1.621452 | 2.610264 | 2.87E-09 |
| AC084876.1 | 2.515676 | 1.935463 | 3.269825 | 5.34E-12 |
| AL360181.2 | 1.573416 | 1.291783 | 1.916451 | 6.66E-06 |
| AC092118.2 | 2.093176 | 1.544391 | 2.836968 | 1.92E-06 |
| AC010201.1 | 2.197782 | 1.653149 | 2.921844 | 5.97E-08 |
| AL513327.1 | 1.583336 | 1.210368 | 2.071233 | 0.000799 |
| AC120053.1 | 2.045284 | 1.522018 | 2.748447 | 2.08E-06 |
| RUSC1-AS1 | 1.70713 | 1.345132 | 2.166547 | 1.09E-05 |
| AC135050.3 | 1.55813 | 1.310364 | 1.852744 | 5.19E-07 |
| AL021707.6 | 1.541108 | 1.286312 | 1.846373 | 2.72E-06 |
| AC243960.1 | 1.713227 | 1.323421 | 2.21785 | 4.36E-05 |
| TMEM147-AS1 | 2.10355 | 1.606286 | 2.754754 | 6.52E-08 |
| LINC00174 | 1.719065 | 1.385504 | 2.13293 | 8.54E-07 |
| LINC02062 | 1.975272 | 1.450737 | 2.689459 | 1.54E-05 |
| MRPL20-DT | 2.705097 | 2.000066 | 3.658653 | 1.05E-10 |
| AL513218.1 | 2.475928 | 1.882062 | 3.257181 | 9.21E-11 |
| AL117379.1 | 1.99013 | 1.585632 | 2.497817 | 2.91E-09 |
