# Supplementary figures and images for: Exploration of Prognostic Immune-Related Genes and lncRNAs Biomarkers in Kidney Renal Clear Cell Carcinoma and Its Crosstalk with Acute Kidney Injury
Source: J Oncol. 2022 Feb 8;2022:6100187. doi: 10.1155/2022/6100187 (PMC8847043; doi:10.1155/2022/6100187)

# Volcano Plot

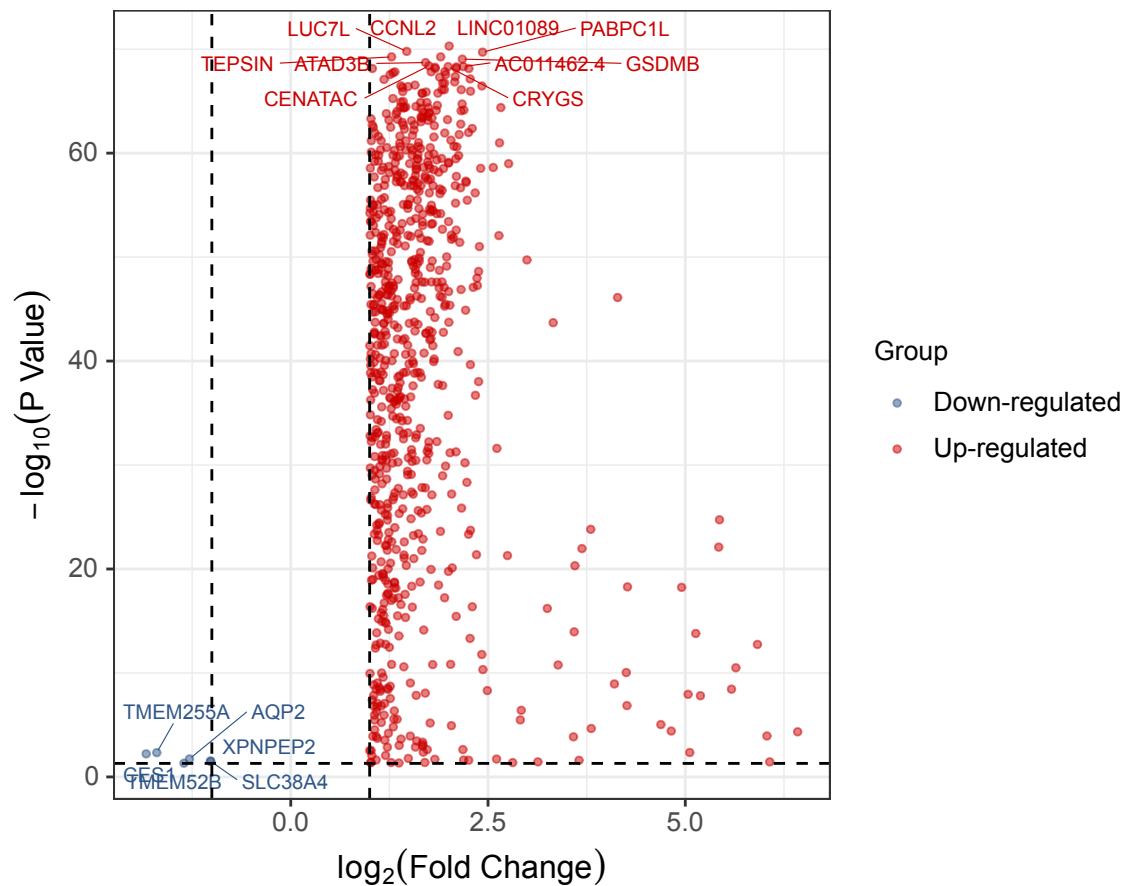

Supplement: Supplementary Materials — Table S1: 2683 IRGs from ImmPort Shared Data. Table S2 : IRGs in the red module. Table S3 : IRGs in the grey module. Table S4: 63 prognostic IRGs. Table S5 : 206 prognostic IR-lncRNAs. Figure S1 : volcano plot showing 765 DEGs between high- and low-risk groups. Figure S2: 44 shared DEGs between KIRC and AKI. [file 6100187.f1.zip › 6100187.f1/Figure S1.pdf]
